# Antibacterial Effect of Autologous Platelet-Rich Gel Derived from Subjects with Diabetic Dermal Ulcers In Vitro

**DOI:** 10.1155/2013/269527

**Published:** 2013-02-28

**Authors:** Lihong Chen, Chun Wang, Hengchuan Liu, Guanjian Liu, Xingwu Ran

**Affiliations:** ^1^Diabetic Foot Care Center, Department of Endocrinology and Metabolism, West China Hospital, Sichuan University, Guoxue Lane No. 37, Chengdu, Sichuan 610041, China; ^2^Teaching and Research Section of Medical Laboratory, West China School of Public Health, Sichuan University, Chengdu, Sichuan 610041, China; ^3^Chinese Evidence-Based Medicine Centre Chinese Cochrane Centre, West China Hospital, Sichuan University, Guoxue Lane No. 37, Chengdu, Sichuan 610041, China

## Abstract

*Background*. Autologous platelet-rich gel (APG) is an effective method to improve ulcer healing. However, the mechanisms are not clear. This study aimed to investigate the antibacterial effect of APG in vitro. *Methods*. Platelet-rich plasma (PRP), platelet-poor plasma (PPP) and APG were prepared from whole blood of sixteen diabetic patients with dermal ulcers. Antibacterial effects against *Staphylococcus aureus*, *Escherichia coli*, and *Pseudomonas aeruginosa* were evaluated by bacteriostasis assay of APG, PRP, and APG-APO (APG combined with apocynin), with phosphate-buffered saline (PBS) and PPP as the control group. *Results*. (1) Compared to the PBS and PPP, the APG and APG-APO groups showed strong antibacterial activity against *Staphylococcus aureus*. There was no significant difference (*P* > 0.05) between APG and APG-APO. (2) Compared to PBS, APG, APG-APO, and PRP showed obvious antibacterial effects against *Escherichia coli* and *Pseudomonas aeruginosa*. No significant difference (*P* > 0.05) was revealed among the three groups. Compared to the PPP group, they did not show antibacterial effect against *Escherichia coli* and *Pseudomonas aeruginosa *(*P* > 0.05). *Conclusions*. APG has antibacterial effect against *Staphylococcus aureus* mediated by platelet activation in the diabetic patients with dermal ulcer, and does not present obvious antibacterial effect against *Escherichia coli* or *Pseudomonas aeruginosa*. Combination of APG and antibiotics may have synergistic antibacterial effect.

## 1. Introduction 

Autologous platelet-rich gel (APG), prepared from whole blood, is a mixture of platelet-rich plasma (PRP), calcium, and thrombin. APG has been used to treat refractory diabetic dermal ulcers for several years. And it turns out to be effective in improving the healing of ulcers [1, 2]. Risk of infection decreases after the use of APG on surgical wounds, in addition to its effect on facilitating healing [3]. One study has revealed the antibacterial activity of APG against *Staphylococcus aureus* [4], and similar results have been shown in our previous research [5]. But in previously published studies outcomes about antibacterial activities of APG against other bacteria (such as *Klebsiella pneumoniae*, *Enterococcus faecalis*, and *Pseudomonas aeruginosa*) are different. And no research has revealed the effects of APG from diabetic ulcer patients. The purpose of the study is to investigate the antibacterial activity of APG (from diabetic patients with dermal ulcers) against *Staphylococcus aureus*, *Escherichia coli*, and *Pseudomonas aeruginosa* in vitro.

## 2. Materials and Methods

### 2.1. Patients

Sixteen patients with diabetic dermal ulcers, 11 men and 5 women, with an average (mean ± SD) age of 61 ± 10 years, were enrolled. The mean duration of diabetes was 8 ± 4 years. The mean HbA1c was 8.8 ± 2.2%. Antibiotics were used intravenously because of clinical importance, and peripheral venous blood was drawn about 8 to 12 hours after antibiotics were used the last time. This study was approved by the ethics committee of West China-hospital. Informed consent was signed and obtained from all subjects.

### 2.2. Preparation of APG

EDTA disodium salt anticoagulated whole blood was obtained from the 16 subjects. Following centrifugation at 313 ×g for 4 minutes, erythrocyte concentrate was removed. PRP and PPP were prepared by centrifugation (1252 ×g) for 6 minutes from the remaining plasma. Thrombin (Heilongjiang Dilong Pharmaceutical Co., China) and calcium gluconate (Sichuan Beauty Sport HuaKang Pharma Co., China) were added to PRP; the gel-like mixture is called APG [6].

### 2.3. Determination of Platelet and Leukocyte Counts

Platelet and leukocyte counts were measured in samples of whole blood and PRP. Platelet enrichment degree [7] was calculated to evaluate the efficiency of the PRP production.

### 2.4. Evaluation of Antibacterial Activity

*Staphylococcus aureus* (ATCC6538), *Escherichia coli* (ATCC8099), and *Pseudomonas aeruginosa* (ATCC15422) (provided by West China School of Public Health, Sichuan University, China) were used to evaluate the antibacterial activity of APG. Bacteria incubated for 24 hours were diluted with sterile PBS; final bacterial count was 10^7^ colony-forming units (CFU)/mL.

Experimental samples were divided into six groups: Group 1 (APG), Group 2 (APG-APO), Group 3 (PRP), Group 4 (PPP), Group 5, and Group 6 (PBS) (Table 1). The APG-APO group was added with apocynin (Sigma-Aldrich Co., USA) to block the possible antibacterial activity of leukocyte [7]. In the PRP group, no thrombin was added to activate the platelets. The PPP group was designed to exclude the antibacterial activity of antibiotics used before when compared to the APG and APG-APO groups. The 5th and 6th groups (PBS1 and PBS2) were designed as the double-blank control. The final bacterial count in each tube was 10^6^ CFU/mL.

After 0, 1, 4, 6, 8, 12, and 24 h, a 0.05 mL sample was taken from each tube. Serial 10-fold dilutions of each sample were made, and 20 uL samples were plated on Mueller-Hinton plates (Beijing Land Bridge Tech Co., China). After 24 h incubation at 37°C, the number of viable bacteria was determined.

The antibacterial rate was calculated using the following formula [5]:

(1) antibacterial rate  =  bacteria counts−bacteria counts in control groupbacteria counts in control group.

### 2.5. Statistical Analysis

The data are reported as mean ± standard deviation. Analysis was performed in SPSS 13.0, using repeated measures analysis for antibacterial activity and one-way ANOVA followed by Tukey's test for platelet count. A value of *P* < 0.05 indicates statistical significance.

## 3. Results

### 3.1. Platelet and Leukocyte Counts

The average volume of blood obtained from subjects was 45 mL, and an average of 4.5 mL PRP was harvested. The platelet count was (242.56 ± 72.33) × 10^9^/L in whole blood, while (1968.8 ± 874.58) × 10^9^/L in PRP with an average 8.1-fold enrichment of platelet concentration after the processing (*P* < 0.05), about (78.56 ± 20.79)% platelet enrichment degree. The leukocyte count of PRP was (5.75 ± 1.46) × 10^9^/L, similar to (5.58 ± 5.89) × 10^9^/L in whole blood.

### 3.2. Antibacterial Activity

#### 3.2.1. Bacterial Counts of *Staphylococcus aureus* over 24 Hours

**Figure 1:**
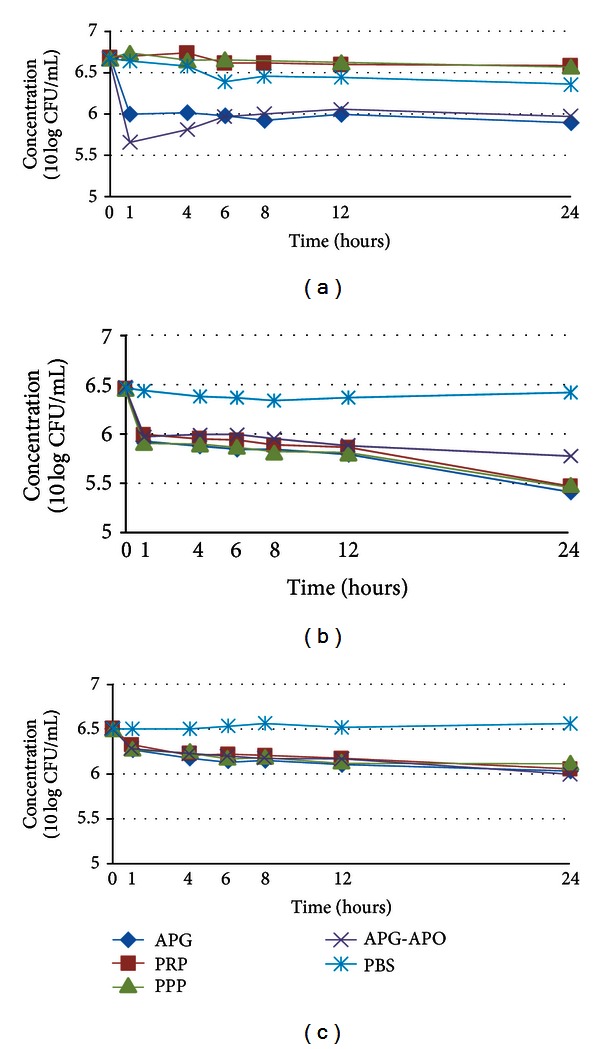
Effect of APG on the counts of various bacteria ((a) *Staphylococcus aureus*; (b) *Escherichia coli*; (c) *Pseudomonas aeruginosa*). APG: autologous platelet-rich gel; APG-APO: APG combined with apocynin; PRP: platelet-rich plasma; PPP: platelet-poor plasma; PBS: phosphate-buffered saline.

Bacterial counts in the APG and APG-APO groups showed a rapid and pronounced decrease compared to PBS group in the first 4 hours (*P* < 0.05) and were still lower than the PBS group in the following 6 hours, although no significant difference was noted (*P* > 0.05). Compared to PRP and PPP groups, bacteria counts in the APG and APG-APO groups reduced significantly (*P* < 0.05) during 24 hours. There were no statistical differences between the APG and APG-APO groups (*P* > 0.05) and between the PRP and PPP groups (*P* > 0.05) (Figure 1(a)).

#### 3.2.2. Bacterial Counts of *Escherichia coli* over 24 Hours

Compared to PBS group, bacterial counts in the APG, APG-APO, PRP, and PPP groups significantly decreased (*P* < 0.05), while no statistical difference was shown among the four groups (*P* > 0.05) (Figure 1(b)).

#### 3.2.3. Bacterial Counts of *Pseudomonas aeruginosa* over 24 Hours

Similar to results of *Escherichia coli*, bacterial counts of *Pseudomonas aeruginosa* in the APG, APG-APO, PRP, and PPP groups reduced significantly (*P* < 0.05) compared to the PBS group, while no statistical difference was shown among the four groups (*P* > 0.05) (Figure 1(c)).

#### 3.2.4. Antibacterial Rate

**Figure 2:**
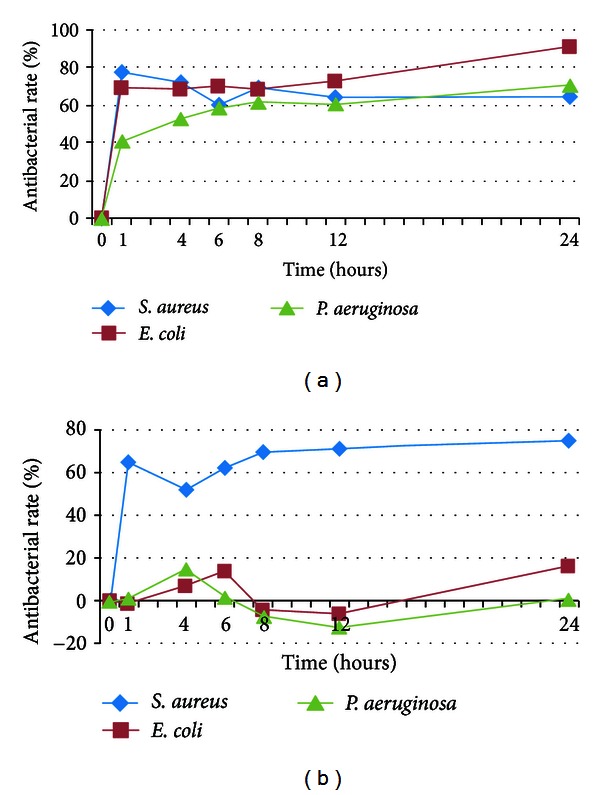
Antibacterial rate of APG against *Staphylococcus aureus*, *Escherichia coli*, and *Pseudomonas aeruginosa* ((a) compared to PBS group; (b) compared to PPP group). *S. aureus*: *Staphylococcus aureus*; *E. coli*: *Escherichia coli*; *P. aeruginosa*: *Pseudomonas aeruginosa*.

Compared to PBS group, the antibacterial rate of APG against *Staphylococcus aureus* reached 77% in the first 4 hours and slowly declined to 61% at the 24th hour. The antibacterial rate against *Escherichia coli* was between 61% and 91%. APG showed lower antibacterial activity against *Pseudomonas aeruginosa* with antibacterial rate of 41% to 70% (Figure 2(a)).

Compared to PPP group, the antibacterial rate of APG against *Staphylococcus aureus* fluctuated between 52% and 77% over 24 hours, while the antibacterial rate of APG against *Escherichia coli* and *Pseudomonas aeruginosa* never reached 20% over 24 hours (Figure 2(b)).

## 4. Discussion

Antibacterial activity of APG against *Staphylococcus aureus* was revealed in this study. And similar effect of APG-APO is also found. This result is consistent with our previous study [5]. In addition, studies from Moojen et al. [4], Bielecki et al. [8], and Isaly and Beckley [9] indicate the similar antibacterial activity against *Staphylococcus aureus*. Compared to PRP, PPP almost does not have erythrocytes, leukocytes, and platelets, while other plasma components (such as proteins and antibiotics used) are similar to the PRP. In order to exclude the antibacterial activity of antibiotics used, the PPP group was designed as a positive control. Furthermore, apocynin was used to block the possible act of leukocytes on bacteria in this study. In this study, we found that there was obvious antibacterial activity of APG when compared with the PPP group. Similar antibacterial activity of APG and APG-APO makes us conclude that the antibacterial effect is not derived from leukocytes. While compared to PRP, the antibacterial effect of APG is still profound. Because the components in APG and PRP are largely the same, except the extra added thrombin and calcium in APG, there is a reason for the contribution of the antibacterial activity to the activation of platelets.

While considering the effect against *Escherichia coli* and *Pseudomonas aeruginosa*, we revealed that similar antibacterial activity is found in the PRP, PPP, APG, and APG-APO groups when compared with the PBS group. Because of the negligible amount of platelets in PPP, the antibacterial activity of PRP, APG, and APG-APO cannot be attributed to the activated platelets and may be contributed to the antibiotics used before.

In fact, platelets play an important part in host-defense system. The abnormality of quantity and quality of platelets can exacerbate infection and increase its related mortality [10–14]. Thrombin, a strong agonist of platelets, was added in the processing of APG. Although the antibacterial mechanisms of APG are not clear, platelets may be play a role. Activated platelets could not only release various growth factors [7] that play an important role in improving the healing of ulcers, but also secret platelet microbicidal proteins (PMPs) [15]. PMPs contain a series of materials which have antibacterial activity, including platelet factor 4, regulated upon activation of normal T-cell expressed and secreted protein, connective tissue-activating peptide 3, platelet basic protein, thymosin beta-4, fibrinopeptide A, and fibrinopeptide B. PMPs could possibly play a role through the following mechanisms: contacting the bacterial membrane, changing the membrane permeability, entering the cell, and inhibiting the synthesis of big molecules [15].

In conclusion, antibacterial activity of APG against *Staphylococcus aureus* was further confirmed, and the effect may be attributed to the activation of platelets in APG. The effect of APG against *Escherichia coli* and *Pseudomonas aeruginosa* comes probably from previously used antibiotics. Therefore, APG itself may have no antibacterial activity against the two bacteria.

However, it is worth noting that the study in vitro is not as the same that as we meet in clinics, such as hyperglycemia, wound repair damage, and immunology change in diabetic patients with severe foot ulcers. Although decreased infection rate was observed by Kachel [3], more research of the antibacterial activity of APG in vivo should be done.

So far, the antibacterial mechanisms of APG are not clear; further investigation is needed to elucidate them.

## Figures and Tables

**Table 1:** Components of each group.

Content (mL)	1	2	3	4	5	6
	APG	APG-APO	PRP	PPP	PBS 1	PBS 2
Bacteria	0.1	0.1	0.1	0.1	0.1	0.1
PRP	0.5	0.5	0.5	—	—	—
PPP	0.4	—	0.45	0.95	—	—
Thrombin-calcium	0.05	0.05	—	—	—	—
Apocynin	—	0.4	—	—	—	—
PBS	—	—	—	—	0.95	0.95

## References

[1] Saldalamacchia G, Lapice E, Cuomo V (2004). A controlled study of the use of autologous platelet gel for the treatment of diabetic foot ulcers. *Nutrition, Metabolism and Cardiovascular Diseases*.

[2] Driver VR, Hanft J, Fylling CP, Beriou JM (2006). A prospective, randomized, controlled trial of autologous platelet-rich plasma gel for the treatment of diabetic foot ulcers. *Ostomy Wound Management*.

[3] Kachel E, Callum J, Moussa F, Goldstein J, Fremes S (2010). Treatment of deep sternal wound infections after coronary artery bypass grafting by means of injection of platelet gel: an evolving technology. *Journal of Thoracic and Cardiovascular Surgery*.

[4] Moojen DJF, Everts PAM, Schure RM (2008). Antimicrobial activity of platelet-leukocyte gel against staphylococcus aureus. *Journal of Orthopaedic Research*.

[5] Yang Y, Liu H, Liu G, Ran X (2010). Antibacterial effect of autologous platelet-rich gel derived from health volunteers in vitro. *Zhongguo Xiu Fu Chong Jian Wai Ke Za Zhi*.

[6] Yuan N, Ran X (2007). Application of autologous platelet-rich gel to refractory chronic diabetic cutaneous ulcers. *Zhongguo Xiu Fu Chong Jian Wai Ke Za Zhi*.

[7] Yuan N, Wang C, Wang Y (2008). Preparation of autologous platelet-rich gel for diabetic refractory dermal ulcer and growth factors analysis from it. *Zhongguo Xiu Fu Chong Jian Wai Ke Za Zhi*.

[8] Bielecki TM, Gazdzik TS, Arendt J, Szczepanski T, Król W, Wielkoszynski T (2007). Antibacterial effect of autologous platelet gel enriched with growth factors and other active substances: an in vitro study. *Journal of Bone and Joint Surgery B*.

[9] Isaly JN, Beckley P An in-vitro determination of platelet gel efficacy as prevention of post-operative bacterial infections. http://www.prpcentral.com/pdf/Related%20Articles/107-Isaly.pdf.

[10] Santolaya ME, Alvarez AM, Avilés CL (2002). Prospective evaluation of a model of prediction of invasive bacterial infection risk among children with cancer, fever, and neutropenia. *Clinical Infectious Diseases*.

[11] Feldman C, Kallenbach JM, Levy H, Thorburn JR, Hurwitz MD, Koornhof HJ (1991). Comparison of bacteraemic community-acquired lobar pneumonia due to streptococcus pneumoniae and klebsiella pneumoniae in an intensive care unit. *Respiration*.

[12] Chang FY, Singh N, Gayowski T (2000). Thrombocytopenia in liver transplant recipients: predictors, impact on fungal infections, and role of endogenous thrombopoietin. *Transplantation*.

[13] Sullam PM, Frank U, Yeaman MR, Tauber MG, Bayer AS, Chambers HF (1993). Effect of thrombocytopenia on the early course of streptococcal endocarditis. *Journal of Infectious Diseases*.

[14] Sun H, Wang X, Degen JL, Ginsburg D (2009). Reduced thrombin generation increases host susceptibility to group A streptococcal infection. *Blood*.

[15] Tang YQ, Yeaman MR, Selsted ME (2002). Antimicrobial peptides from human platelets. *Infection and Immunity*.

